# Comparing the Efficacy of Two Internet-Based, Computer-Tailored Smoking Cessation Programs: A Randomized Trial

**DOI:** 10.2196/jmir.7.1.e2

**Published:** 2005-03-08

**Authors:** Jean-François Etter

**Affiliations:** ^1^Institute of Social and Preventive MedicineUniversity of GenevaGenevaSwitzerland

**Keywords:** Tobacco dependence, Internet, randomized controlled trials, smoking cessation, behaviour change

## Abstract

**Background:**

Online computer-tailored smoking cessation programs have not yet been compared directly.

**Objective:**

To compare the efficacy of two Internet-based, computer-tailored smoking cessation programs.

**Methods:**

Randomized controlled trial conducted in 2003-2004. Visitors to a smoking cessation website were randomly assigned to either an original online, interactive smoking cessation program or to a modified program. Both programs consisted of tailored, personalized counseling letters based on participants' characteristics, followed by monthly email reminders. The original program was based on psychological and addiction theory, and on preliminary research conducted in the same population. The modified program was shorter and contained more information on nicotine replacement therapy and nicotine dependence, and less information on health risks and coping strategies. In both programs, 1 month and 2 months after entering the study, participants were invited by email to answer the same tailoring questionnaire again in order to receive a second counseling letter. Participants in both programs obtained, on average, 1.2 feedback counseling letters over 2.5 months, and 84% received only 1 feedback letter. The outcome was self-reported smoking abstinence (no puff of tobacco in the previous 7 days), assessed 2.5 months after entry in the program. We report results from intention-to-treat (ITT) analyses, where all non-respondents at follow-up were counted as smokers.

**Results:**

The baseline questionnaire was answered by a total of 11969 current (74%) and former (26%) smokers, and the follow-up survey by 4237 people (35%). In an ITT analysis, abstinence rates in baseline current smokers were respectively 10.9% and 8.9% (odds ratio [OR]=1.24, 95% confidence interval [CI]1.08-1.43, *P*=.003) in the original and modified programs, and 25.2% and 15.7% (OR=1.81, CI 1.51-2.16, *P*<.001) in baseline former smokers. While we found statistically significant differences in quit rates in smokers in the contemplation stage favoring the original program (OR=1.54, CI 1.18-2.02, *P*=.002), no between-group differences in quit rates were observed in smokers in the precontemplation (OR=1.07, CI 0.36-3.14, *P*=.91) and preparation (OR=1.15, CI 0.97-1.37, *P*=.10) stages of change.

**Conclusions:**

In smokers in the contemplation stage of change and in former smokers, the original program produced higher smoking abstinence rates than the modified program.

## Introduction

Self-help smoking cessation booklets and leaflets can reach large numbers of smokers, but they may not be very effective [1]. Computer technology and psychological theory can be combined to produce effective individualized self-help smoking cessation materials and to disseminate them at the population level, in particular on the Internet [2]. Because individually tailored materials take into account the relevant characteristics of each participant, smokers may be more interested in reading these materials than untailored booklets, and may be more likely to apply the advice included therein [3,4]. Consequently, tailored materials may be more effective than those intended for all smokers [1,5]. Several studies have tested the effectiveness of computer-tailored smoking cessation programs, with positive and negative results [1,6]. These programs were evaluated either on personal computers or when feedback materials were printed and sent by mail. Few studies tested the effect of smoking cessation programs administered on the Internet [7,8,9]. An early randomized trial conducted in 1998 on CompuServe showed that after 3 months smoking abstinence rates were higher in smokers who took part in an online discussion group and received e-mail messages compared to a control group, but this effect was not maintained at 6-month follow-up [7]. More recently, the only other randomized trial on this topic showed that in nicotine patch users, an online computer-tailored program was more effective in the short-term (10 weeks) than a non-tailored program [9]. A non-randomized trial showed that the effect of an online interactive program could be improved by tailored follow-up by email [8]. We know of no randomized trial comparing two online, computer-tailored smoking cessation programs. Such comparisons are nevertheless necessary, given the large variability in the effect of these programs [1].

In a previous study, we tested a paper version of a computer-tailored program [10,11]. In this version, questionnaires, computer-tailored counseling letters and stage-matched booklets were sent by mail to smokers. This study showed that 7 months after entry into the program, 7-day smoking abstinence rates were 2.4 times greater (8.0% vs 3.3%, *P*<.001) in the intervention group than in a control group that received no treatment. The same program is also available online, but the efficacy of the online version is unknown. We compared the online version of this program with another online smoking cessation program intended for users of nicotine replacement products.

Several websites offer interactive, computer-tailored smoking cessation programs, but these programs have never been compared directly in a randomized trial. Our aim was to compare the efficacy of two online, computer-tailored smoking cessation programs.

## Methods

### Setting and Participants

Participants were visitors of Stop-tabac.ch, a French-language website that provides information, advice, and support to smokers and ex-smokers. This website was listed among the 5 best websites on smoking cessation in a recent study [12], and it is listed first in Google.fr when searching with the words *arrêter de fumer*, *fumer*, or *tabac* (quit smoking, smoke, or tobacco) (tested February 21, 2005).

### Interventions

Various services are available to visitors of Stop-tabac.ch, including fact sheets, booklets, answers to frequently asked questions, personal stories written by current and former smokers, discussion forums and chat rooms, tests, games, and two interactive, computer-tailored smoking cessation programs [10,11]. Each month, about 2% of the 50000 monthly visitors of the website take part in these interactive computer-tailored programs [13]. After reading an information page that briefly describes the programs, participants are informed that they will have to answer a questionnaire, that their answers will be retained on file, and that the data will be used only to organize a follow-up and for statistical analyses conducted in an anonymous format. They have the option of refusing to have their answers retained on file. The next step consists of answering the tailoring questionnaire. Enrollment of participants in this study took place between April 2003 and July 2004. In this period, two different questionnaire forms, referring participants either to the original or the modified program, appeared alternatively in random order. Thus participants were randomly assigned to either program.

Both programs consisted of tailored personal counseling letters compiled by a computer according to the answers made by participants. The counseling letters appeared on the screen immediately (<5 seconds) after the answers were submitted. Participants were advised to print their counseling letter and to read it again later. Participants in the original program were also advised to print stage-matched booklets available on the website.

### The Original Program

**Figure 1:**
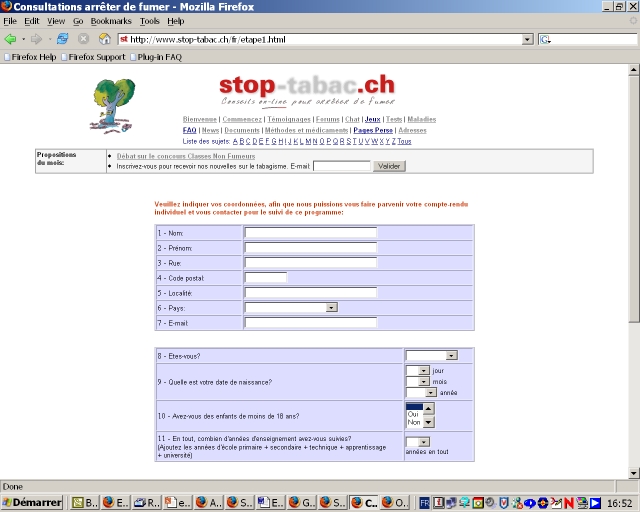
Screenshot of the tailoring questionnaire for the original program

**Figure 2:**
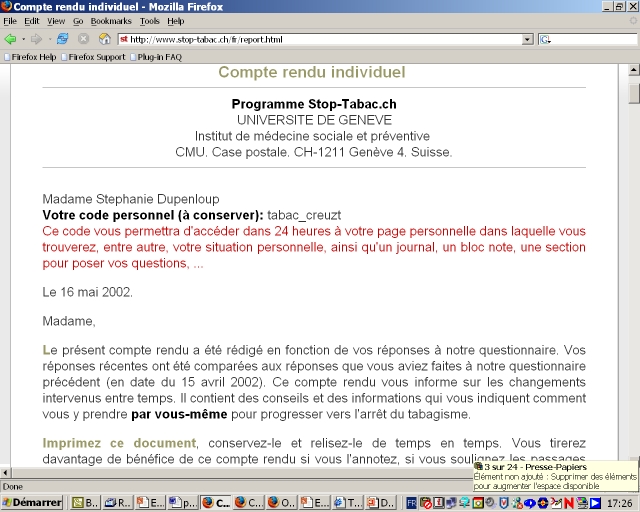
Personal counseling letter of the original program

The original program was based on the Transtheoretical Model of Change [14,15], on the Theory of Planned Behavior [16], on theories of relapse prevention [17] and tobacco dependence [18], on the Agency for Health Care Policy and Research recommendations [19], and on relevant literature [20,21]. The questionnaire, counseling letters, and brochures were also based on extensive research conducted on Swiss smokers and ex-smokers [22,23,24]. The tailoring questionnaire (Figure 1) assessed demographic characteristics, smoking status, stage of change (*precontemplation*, no intention of quitting smoking in the next 6 months; *contemplation*, seriously considering quitting in the next 6 months; *preparation*, has decided to quit in the next 30 days; *action*, has quit smoking for 6 months or less; and *maintenance*, has quit smoking for more than 6 months)[15], level of tobacco dependence, attitudes towards smoking, self-efficacy, use of self-change strategies and coping methods, and intention to use nicotine replacement therapy (NRT). We used validated multi-item scales to measure these variables [22,23,24,25]. Former smokers indicated the date that they had quit smoking. After answering the 62-item questionnaire, participants received a personal counseling letter of 6 to 9 pages (3000-4000 words) illustrated with cartoons and graphs that were also tailored to each participant's answers (Figure 2). The counseling letters consisted of about 20 paragraphs of text, chosen by the computer in a library of 350 paragraphs according to pre-established decision rules. This program was launched online in French in 1997 and was later expanded to include English, Danish, Italian and Chinese versions [13]. The interactive program was updated to include innovations (eg, new NRT products and bupropion), pictures, and a few additional questions and feedback paragraphs. Overall, the online version of the program tested in the present study is nevertheless largely similar to the paper version tested in our previous studies [10,11].

### The Modified Program

**Figure 3:**
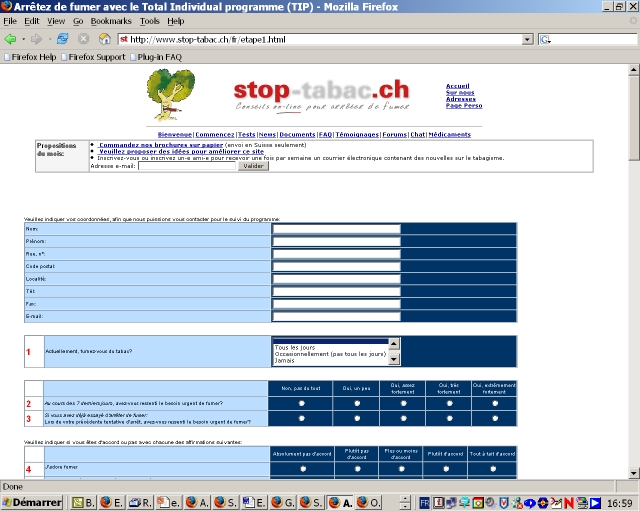
Screenshot of the tailoring questionnaire for the modified program

**Figure 4:**
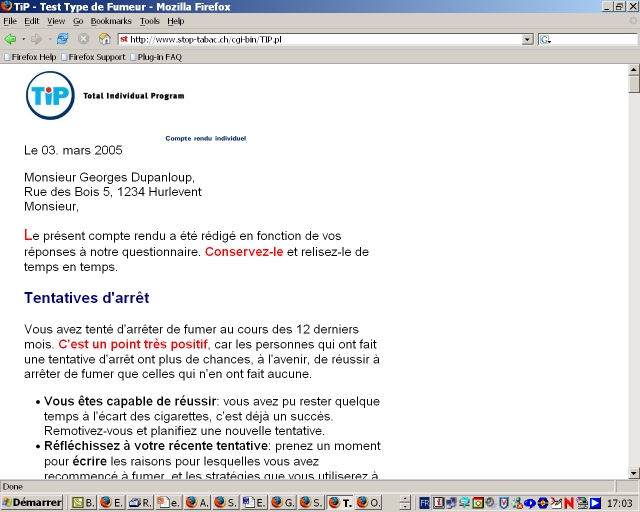
Personal counseling letter of the modified program

The modified program was developed by us for Novartis, a producer of nicotine replacement products, when these products became available over-the-counter (OTC, ie, without a medical prescription) in Switzerland in 2000. This program was intended to provide some smoking cessation counseling to smokers who bought OTC NRT products and thus did not receive medical supervision. Compared with the original program, the modified program used a shorter questionnaire (38 questions) that included ad hoc questions instead of validated multi-item scales (Figure 3). The counseling letter was of similar length (3000-4000 words), but contained more information on NRT and nicotine dependence, and less information on health risks and coping strategies (Figure 4).

### Additional Program Interactions

In both programs, 1 month and 2 months after entering the study, participants were invited by email to answer the same tailoring questionnaire again in order to receive a second counseling letter. To write the second letter, the computer compared each participant's new answers with the answers given on their previous visit. Participants were congratulated for any progress they had made since their last visit or encouraged, if they had relapsed. In both groups, participants received on average 1.2 counseling letters; 84% of participants received only 1 counseling letter and 16% 2 or more letters.

### Outcome Measures

To assess smoking abstinence, an email message was sent out 11 weeks after receipt of the baseline questionnaire; those who failed to respond received up to 3 email reminders. Participants answered the following question by clicking on *Yes* or *No* directly in the email message: "Did you smoke any tobacco in the past 7 days (even one puff of cigarette, cigar, pipe, etc)?" The criterion of 7 days' abstinence was used in a recent guideline to assess smoking cessation in randomized trials [26]. We used an intention-to-treat analysis in which all non-respondents were counted as smokers.

### Sample Size Calculations

Sample size calculations indicated that a sample of 5300 was necessary to detect a between-program difference of 2 percentage points in abstinence rates in current smokers (8% vs 6%, confidence level 95%, power 80%). The expected difference of 2 percentage points was estimated on a basis of a synthesis of previous studies of computer-tailored programs [1], and taking into account an expected follow-up rate of about one third [28] and an intention-to-treat analysis. With its final sample size of 11969 participants, the study was powered to detect differences in subgroups of participants, in particular current and former smokers.

### Statistical Analyses

We used chi-square tests to compare proportions (eg. abstinence rates) and *t* tests to compare means. We used odds ratios (OR's) with 95% confidence intervals (CI's) to express the proportion of non-smokers (abstinence rate) in the original program compared to the proportion of non-smokers in the modified program. We tested the effectiveness of the program in subgroups, stratifying by age, sex, number of cigarettes per day, and stage of change.

Because participation rates in the follow-up survey differed in the two groups, we report both intention-to-treat data, where all baseline participants were included in the denominator and non-respondents were counted as smokers, and an analysis including only those who took part in the follow-up survey. We also conducted a sensitivity analysis, extrapolating results under a hypothetical situation where response rates to the follow-up survey were assumed to be the same in the two study arms.

## Results

### Participation

**Figure 5:**
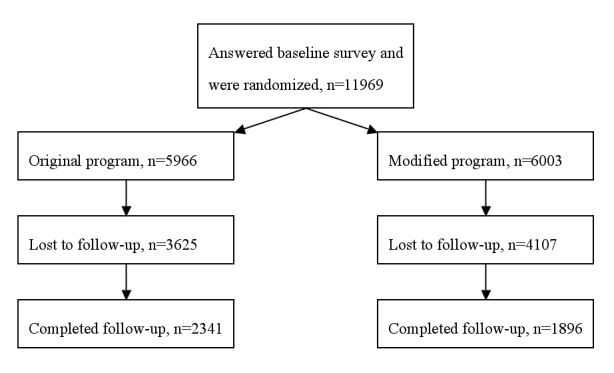
Flow chart of participants in the randomized controlled trial

The raw database included 12434 records. We excluded 245 participants who had taken part in both programs and deleted 220 entries of people registered twice in the same program. Thus 11969 participants were included in the study. Figure 5 illustrates the flow of participants through the trial.

At baseline, the two study groups were similar in terms of age and sex distributions, smoking status (current or former smokers), stage of change, cigarette consumption, and, among former smokers, the number of days since smoking cessation (Table 1). As in a previous study [13], the sample included a substantial proportion of former smokers (n=3095, 26%), and relatively few smokers in the precontemplation stage of change (n=385, 3%). Smokers in this study were more motivated to quit smoking than a representative sample of smokers in Geneva (distribution of smokers by stage of change in Geneva: 74%, precontemplation; 22%, contemplation; 4%, preparation) [27].

**Table 1:** Baseline characteristics of study participants

		**Original Program**	**Modified Program**	**P**
Number of participants		5966	6003	
Age (mean, SD)		34.1	33.8	.13
Men (n, %)		2308 (39.0)	2181 (38.2)	
**Smoking status**				.73
	Current smokers (n, %)	4346 (73.9)	4336 (73.6)	
	Former smokers (n, %)	1538 (26.1)	1557 (26.4)	
**Among current smokers**				
	Cigarettes per day (mean)	19.6	19.3	.06
	Minutes to first cigarette of the day (mean)	50.5	51.3	.64
	Made a quit attempt in the previous year (n, %)	2079 (47.9)	2104 (49.4)	.15
**Stage of change (n, %)**				.13
	Precontemplation	214 (4.9)	171 (4.0)	
	Contemplation	1497 (34.5)	1480 (34.9)	
	Preparation	2623 (60.5)	2584 (61.0)	
**Among former smokers**				
	Interval since quit date (days, mean)	101	91	.73
**Stage of change**				.06
	Action (n, %)	1349 (93.7)	1082 (91.9)	
	Maintenance (n, %)	90 (6.3)	96 (8.1)	

The response rate to the follow-up survey was 35.4% (4237 of 11969). However, more participants in the original program (n=2341, 39.2%) than in the modified program (n=1896, 31.6%) answered the follow-up survey (χ^2^=76.7, *P*<.001). In both groups, the median interval between the baseline and follow-up surveys was 2.5 months (25th, 50th, and 75th percentiles in both groups; 75, 77 and 80 days respectively).

### Smoking Abstinence Rates

At follow-up, when all baseline participants were included in the denominator and non-respondents were counted as smokers, the 7-day abstinence rate was higher for the original program than for the modified program (14.6% vs 10.7%, *P*<.001, OR=1.43, 95% CI 1.28 - 1.59) (Table 2). Thus compared with the modified program, the original program produced 1 additional quitter for every 26 participants. The original program was more effective than the modified program in baseline current smokers (abstinence rates: 10.9% vs 8.9%, OR=1.24, CI 1.08-1.63, *P*=.003) and in baseline former smokers (25.2% vs 15.7%, OR=1.81, CI 1.51-2.17, *P*<.001).

Among smokers in the precontemplation and preparation stages of change, there was no statistically significant difference in quit rates between programs; but the original program produced more quitters than the modified program among smokers in the contemplation stage (Table 2). Among light smokers (1-10 cigarettes/day), there was no difference in quit rates between programs; but the original program was more effective than the modified program in smokers of 11 to 24 cigarettes/day (OR=1.28) and in heavy smokers (25 or more cigarettes/day) (OR=1.54). The relative effect of the two programs was the same in men and women and across age groups. Interestingly, younger smokers (≤19 years old) were the least likely to quit smoking.

### Secondary Analysis

When we included only the 4237 participants who answered the follow-up survey, abstinence rates were significantly (χ^2^=5.0, *P*=.03) higher in the original program (873 out of 2341, 37.3%) than in the modified program (644 out of 1896, 34.0%).

### Sensitivity Analysis

In a sensitivity analysis, we extrapolated data assuming a hypothetical situation where the same proportion of participants in both groups (39.2%) answered the follow-up survey. Under this assumption, 2356 out of 6003 (39.2%) participants in the modified program (instead of 1896) would have answered the follow-up survey, and 800 out of 2356 (34.0%) would have quit smoking. Under this assumption, and including all baseline participants in the denominator, 13.3% (800 out of 6003) would have been abstinent in the modified program versus 14.6% (873 out of 5966) in the original program (χ^2^=4.3, *P*=.04). Thus the original program was still more effective than the modified program, even after taking into account the difference between groups in response rates.

**Table 2:** Smoking abstinence rates (no puff of tobacco smoke in the past 7 days), intention-to-treat analysis, 2.5 months after entry into two computer-tailored smoking cessation programs on a French-language smoking cessation website, 2003-2004

		**n in Analysis**	**Original Program**	**Modified Program**	**Odds Ratio**	**95% confidence interval**	**P**
All participants (N, %)		11969	873 (14.6)	644 (10.7)	1.43	1.27-1.59	<.001
Men		4489	339 (14.7)	235 (10.8)	1.43	1.19-1.70	<.001
Women		7145	534 (14.8)	406 (11.5)	1.34	1.17-1.54	<.001
**Age**							
	<19 years	492	11 (4.5)	6 (2.4)	1.92	0.70-5.28	.20
	20-29	3936	226 (11.5)	186 (9.4)	1.25	1.02-1.53	.03
	30-39	3920	357 (17.5)	238 (12.7)	1.46	1.22-1.74	<.001
	40-49	2259	207 (17.7)	156 (14.3)	1.29	1.03-1.62	.03
	50-77	940	72 (14.9)	46 (10.1)	1.57	1.05-2.31	.03
All current smokers		8682	472 (10.9)	388 (8.9)	1.24	1.08-1.43	.003
**Stage of change at baseline**							
	Precontemplation	385	8 (3.7)	6 (3.5)	1.07	0.36-3.14	.91
	Contemplation	2977	143 (9.6)	95 (6.4)	1.54	1.18-2.02	.002
	Preparation	5207	321 (12.2)	279 (10.8)	1.15	0.97-1.37	.10
**Smoking rate**							
	Very light smokers (1 to 5 cig./day)	308	11 (7.6)	13 (7.9)	0.96	0.42-2.22	.93
	Light smokers (6-10 cig./day)	1385	63 (9.1)	76 (11.0)	0.81	0.57-1.15	.23
	Average smokers (11-24 cig./day)	4520	242 (10.7)	192 (8.5)	1.28	1.05-1.56	.015
	Heavy smokers (> 25 cig./day)	2347	154 (12.9)	101 (8.8)	1.54	1.18-2.01	.001
All former smokers		3095	387 (25.2)	244 (15.7)	1.81	1.51-2.17	<.001
Action stage of change		2305	340 (26.2)	198 (19.6)	1.46	1.19-1.78	<.001
Maintenance stage of change		175	23 (27.4)	19 (20.9)	1.43	0.71-2.87	.31

## Discussion

### Efficacy

We compared two Internet-based computer-tailored smoking cessation programs: a program based on theory and preliminary research conducted in the study population; and a modified and simplified version of the same program designed for NRT users. The original program was more effective than the modified program in helping current smokers in the contemplation stage of change quit smoking and in helping former smokers avoid relapse. In a previous study, we showed that when implemented on paper (ie, when counseling letters and booklets were sent by mail), the original program was more effective than no intervention [10,11]. This study showed that the efficacy of this program was apparently maintained when it was implemented over the Internet. Among baseline smokers, 7-day abstinence rates were quite comparable in the Internet version (10.9%) and in the paper version of the original program (8%) [10]. In this previous study, we tested the original program on current smokers only. The present study suggests that this program was also effective in preventing relapse in former smokers.

Because the present study did not include a no-treatment control group, we are unable to say whether both programs were more effective than no intervention. However, quit rates in smokers in the modified program (8.9%) were higher than quit rates in smokers in the no-treatment control group in our previous study (3.3%) [10], which suggests that even the modified program might be more effective than no intervention. Tests of the Internet versions of both programs against a no-treatment control group are nevertheless warranted, but such tests are made difficult by the risk of selective drop-out in the no-treatment group, and by the potential for contamination from external programs, as disappointed participants in the no-treatment group could obtain counseling from other websites able to be found in just a few clicks.

The follow-up in both programs consisted of short, monthly email messages inviting participants to answer the same questionnaire again, in order to receive a second counseling letter that was largely similar to the first one. This follow-up procedure may not have been intensive enough, which may explain why so few participants obtained additional counseling letters. The follow-up in the program could be improved by using individually tailored email messages, sent more frequently just before and after the quit date, as was done by Lenert and colleagues [10].

### Cost

The total cost of implementing the website where the two programs are available is currently 70000 Swiss francs a year (US$ 60000), for a reach of over 8000 participants per year in the computer-tailored programs, and for 600000 visitors per year to the website (where other features, such as discussion forums and personal stories, are more popular than the computer-tailored program). The average duration of a visit to the website is 7 minutes, with an average of 8 pages viewed per visit. This is comparable to the cost of running a small smoking cessation clinic which would treat about 50 smokers a month.

### Strengths and Limitations of This Study

A strength of this study is that it was powered to detect small differences in quit rates in subgroups of participants (eg, current and former smokers). The response rate at follow-up was low (35%), but it was close to the average response rate of 39.6% reported in a meta-analysis of 68 Internet-based surveys [28], and it was in the range of 30-40% in response rates obtained in follow-up surveys of the three other efficacy trials of online smoking cessation programs [7,8,9]. Follow-up rates in Internet studies are lower than those usually found in smoking cessation studies. Several steps could be taken to increase follow-up rates in Internet surveys such as: asking participants to indicate a second email address or the email address of a relative; asking them to keep their email address active for the duration of the study; requiring participants to commit to taking part in the follow-up survey; and asking for a phone or fax number, or a postal address. Paying participants could introduce bias and is not a very cost-effective option, given the large samples obtained in Internet studies.

There were more non-respondents in the modified program group than in the original program. This could produce an artificial advantage for the original program in intention-to-treat analyses where non-respondents are counted as smokers. However, even when data were analyzed in respondents only, quit rates were higher in the original program. In addition, a sensitivity analysis showed that if participation rates had been similar in both study arms, the original program would still have been more effective than the modified program. Under this assumption however, the between-group difference would have been smaller.

Fewer participants in the modified program than in the original program took part in the follow-up. The modified program was developed in collaboration with a pharmaceutical company and emphasized NRT use. Participants were informed of this collaboration and may have been less keen to take part in the follow-up of a program associated with the industry than in the original program, which was university based.

Because the study did not include a no-treatment control group, it remains possible that the natural quit rates in this sample (ie, the quit rate outside any intervention) lies somewhere between the quit rates measured in the 2 study arms. In this case, the programs would have no effect. However, the original program produced similar quit rates whether it was implemented on the Internet or on paper, and these quit rates were higher that in the no-treatment control group in our previous study [10]. This suggests that the original program is more effective than no intervention. Nevertheless tests of the online versions of both programs against no-intervention control groups are warranted.

The difference in program efficacy between the original and modified versions was observed only in smokers in the contemplation stage of change, but not in those in the precontemplation and preparation stages. Similarly, the paper version of the original program had no effect in smokers in the preparation stage [10]. The paper version had however a significant impact in smokers in the precontemplation (3 percentage points) and contemplation stages (4 percentage points) [10]. These results suggest that this program may be effective mainly in motivating contemplators to make a quit attempt. A new version of the program should be developed in order to better take into account the needs of smokers in the preparation stage.

We measured point prevalence of abstinence after 2.5 months, but this approach may not reflect long-term continuous abstinence rates. In our previous study of the paper version of the original program, we showed that the effect measured 7 months after entry into the program was not maintained, one-and-a-half years after the intervention was stopped [11]. Previous research showed that one half of the people who succeed in abstaining from smoking for 6 months will relapse within 5 years [29]. Thus long-term follow-up studies are needed to assess whether Internet-based programs have sustained effects. The only existing studies are short-term (<=6 months) [7, 8,9]. Long-term studies are however limited by the difficulty of obtaining high response rates in Internet surveys [28].

We conducted no biochemical verification of smoking status for several reasons. First, collecting saliva samples for the determination of cotinine (a metabolite of nicotine) or collecting expired carbon monoxide would have decreased participation rates [30]. Second, biochemical verification will not change the results of most smoking cessation studies because self-report is generally accurate in adults, and because large between-group differences in misreporting are unlikely [31]. Third, biochemical verification is not recommended in large scale population-based studies with limited face-to-face contact, and in studies where data collection is done over the Internet [32]. In a study conducted in a similar population, we showed that for the association between saliva cotinine and self-report of smoking, the area under the receiver operating characteristic curve was 0.95, and that most cases of disagreement were due to occasional smokers rather than to misreporting [33]. Furthermore, at least two studies indicated that in intervention trials, self-report of smoking was not, or only minimally, biased in intervention groups, compared with controls [34,35]; therefore, such bias would not explain away our results.

### Conclusion

The original program was more effective than a modified version of the same program intended for NRT users. Given the already documented large variability in the effect of computer-tailored programs [1], other available online smoking cessation programs should be compared directly, in randomized trials [36].

## Abbreviations

CI: Confidence Interval
ITT: Intention-To-Treat
NRT: Nicotine Replacement Therapy
OR: Odds Ratio
OTC: Over-the-Counter
